# Intravaginal Practices, Vaginal Infections and HIV Acquisition: Systematic Review and Meta-Analysis

**DOI:** 10.1371/journal.pone.0009119

**Published:** 2010-02-09

**Authors:** Adriane Martin Hilber, Suzanna C. Francis, Matthew Chersich, Pippa Scott, Shelagh Redmond, Nicole Bender, Paolo Miotti, Marleen Temmerman, Nicola Low

**Affiliations:** 1 Institute of Social and Preventive Medicine, University of Bern, Bern, Switzerland; 2 Infectious Disease Epidemiology Unit, London School of Hygiene and Tropical Medicine, London, United Kingdom; 3 Faculty of Medicine and Health Sciences, International Center for Reproductive Health, Ghent University, Ghent, Belgium; 4 Reproductive Health and HIV Research Unit, University of Witwatersrand, Johannesburg, South Africa; 5 Office of AIDS Research, United States National Institutes of Health, Bethesda, Maryland, United States of America; The Royal College of Physicians and Surgeons of Canada, Canada

## Abstract

**Background:**

Intravaginal practices are commonly used by women to manage their vaginal health and sexual life. These practices could, however, affect intravaginal mucosal integrity. The objectives of this study were to examine evidence for associations between: intravaginal practices and acquisition of HIV infection; intravaginal practices and vaginal infections; and vaginal infections and HIV acquisition.

**Methodology/Principal Findings:**

We conducted a systematic review of prospective longitudinal studies, searching 15 electronic databases of journals and abstracts from two international conferences to 31^st^ January 2008. Relevant articles were selected and data extracted in duplicate. Results were examined visually in forest plots and combined using random effects meta-analysis where appropriate. Of 2120 unique references we included 22 publications from 15 different studies in sub-Saharan Africa and the USA. Seven publications from five studies examined a range of intravaginal practices and HIV infection. No specific vaginal practices showed a protective effect against HIV or vaginal infections. Insertion of products for sex was associated with HIV in unadjusted analyses; only one study gave an adjusted estimate, which showed no association (hazard ratio 1.09, 95% confidence interval, CI 0.71, 1.67). HIV incidence was higher in women reporting intravaginal cleansing but confidence intervals were wide and heterogeneity high (adjusted hazard ratio 1.88, 95%CI 0.53, 6.69, I^2^ 83.2%). HIV incidence was higher in women with bacterial vaginosis (adjusted effect 1.57, 95%CI 1.26, 1.94, I^2^ 19.0%) and *Trichomonas vaginalis* (adjusted effect 1.64, 95%CI 1.28, 2.09, I^2^ 0.0%).

**Conclusions/Significance:**

A pathway linking intravaginal cleaning practices with vaginal infections that increase susceptibility to HIV infection is plausible but conclusive evidence is lacking. Intravaginal practices do not appear to protect women from vaginal infections or HIV and some might be harmful.

## Introduction

Intravaginal practices comprise a variety of behaviors that women use to positively manage their health and sexual life [1]. Women often use these practices to achieve a desired intravaginal state, which they believe will keep them clean and free from disease, and sexually desirable [2]. Vaginal stratified squamous epithelium is an important barrier to infection but physical, chemical, or biological factors associated with intravaginal practices could allow HIV to infect intraepithelial Langerhans cells or be taken up by migratory dendritic cells and disseminated to regional lymph nodes [3]. For example, insertion or application of substances like herbs, pulverized rock, or commercial products to prepare the vagina for sexual intercourse can cause physical or chemical abrasions [4] that could be exacerbated during intercourse [3]. These kinds of practices have previously been referred to as ‘dry sex’ [5], [6]. Wiping out the vagina with cloth, cotton wool or paper during sex [7] or after intravaginal cleansing might have similar effects. Soaps, detergents and antiseptics used to wash inside the vagina can cause chemical damage [8] and increase vaginal pH, encouraging the growth of organisms associated with bacterial vaginosis [9], a condition shown to increase women's risk of HIV infection acquisition in many prospective studies [10]. Cloths used commonly in some countries to clean the vagina repeatedly might also act as fomites, harboring *Trichomonas vaginalis*, which can increase the risk of HIV acquisition [11], [12].

**Figure 1:**
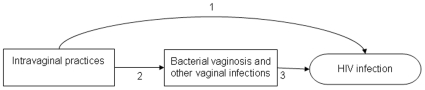
Simplified hypothesized pathways linking intravaginal practices and HIV. Intravaginal practices are hypothesized to cause physical or chemical damage to vaginal epithelium. In response, changes in vaginal flora associated with bacterial vaginosis occur, or colonization by other vaginal pathogens occurs. These conditions facilitate HIV transmission. Intravaginal practices might also increase the transmission of HIV through a direct effect or other pathways. Numbers refer to the objectives of the review. Objective 1 includes the estimation of the association between intravaginal practices and HIV infection. Objective 2 estimates the association between intravaginal practices and vaginal infections. Objective 3 estimates the association between disrupted vaginal flora, bacterial vaginosis or other vaginal infections and HIV-1 acquisition.

Biological and epidemiological data have been integrated to hypothesize a pathway linking intravaginal practices with acquisition of HIV infection, which might be mediated by changes in vaginal flora or vaginal infections that disrupt mucosal integrity (Figure 1) [1], [13], [14]. Results from individual epidemiological studies have been inconclusive [14]–[16]. Differences in definitions and classification of vaginal practices, measures of intensity of exposure, study design, study population, and methods of statistical analysis might explain these inconsistent results [17]. In addition, some intravaginal practices are uncommon and HIV infection is a relatively rare outcome so potentially important associations could be missed. We carried out a systematic review to synthesize epidemiological evidence about the steps along the potential causal pathway (Figure 1). The specific objectives were to examine associations between: 1) intravaginal practices and HIV acquisition; 2) intravaginal practices and vaginal infections; and 3) vaginal infections and HIV acquisition in prospective studies in women.

## Methods

Ethical approval was not required for this review because only published data were included.

### Literature Searches

The authors searched electronic databases from the earliest date up to 31^st^ January 2008. The full search strategy is available on request. In brief, we searched Excerpta Medica Database (EMBASE); Medical Literature Analysis and Retrieval System Online (MEDLINE); Cumulative Index to Nursing and Allied Health Literature (CINAHL); the Cochrane Library; and Educational Resources Information Centre (ERIC) databases using explosion searches for subject headings or thesaurus terms for disease outcomes; HIV, bacterial vaginosis, trichomoniasis, candidiasis. We combined these with free text terms and wildcard characters related to intravaginal practices, including: vagina; vulva; intravaginal; dry sex; cleansing; cleaning; washing; cutting; douching; insertion; practice; lubrication; microbicide and genital lesions. We used the same combinations of free text keywords to search ten other electronic databases including Global Health Library (GHL); Population Information Online (Popline) and WHO regional indexes. We hand-searched conference abstracts from the International AIDS Society Conferences, the Conference on Retroviruses and Opportunistic Infections, the International Society for STD Research, and Microbicides conferences since 1990. Reference lists of included papers, systematic reviews, letters and commentaries were examined, and experts were contacted to identify additional papers. There were no language restrictions.

### Selection of Studies

Eligible studies were prospective longitudinal studies or randomized controlled trials of HIV seronegative women, which reported on intravaginal practices and documented incident HIV seroconversions or episodes of bacterial vaginosis, trichomoniasis or vaginal candidiasis. Case-control studies were included if they were nested within longitudinal studies. Cross-sectional studies, editorials, commentaries, letters without original data and case reports were not included. Articles in languages other than English were translated by the authors.

### Definitions of Intravaginal Practices

We based our definitions on practices identified during a qualitative research study in four countries in sub-Saharan Africa and South East Asia [2]. We considered four eligible categories of intravaginal practice: intravaginal cleansing with liquids (including douching); insertion of dry substances into the vagina; ingestion of substances intended to affect the vagina; and self-administered anatomical modifications, such as cutting at the introitus, with insertion into the cut of traditional substances that are washed away later. We attempted to further define each category of intravaginal practice according to the means of application or delivery and specific products used. External practices involving washing, steaming (or “fogging”) and application of substances onto the labia or vulva were not eligible. We excluded female genital mutilation or elective surgery to alter vaginal anatomy because these are not done by the woman herself. We also excluded use of sex toys, use of female and male condoms and other barrier contraceptives such as the diaphragm or sponge, other devices for delivering of medications, and tampons or other commercial products for the absorption of menstrual blood. Previous qualitative research in southern Africa has found that local African languages have different words for intravaginal and external washing so differences between these practices are understood [2]. We therefore assumed that studies describing vaginal washing, cleansing or insertion meant practices that affected the vaginal mucosa beyond the introitus and not just the vulva.

### Definition of Outcomes

The primary outcome was incident HIV-1 infection, as defined by authors of each study. We defined intermediate vaginal conditions as investigator-defined criteria for: bacterial vaginosis (Nugent score of 7 to 10 [18] or 3 or more Amsel criteria [19]); disturbed vaginal flora (Nugent score 4 to 6); vaginal yeast infections including candidiasis; and *Trichomonas vaginalis*. Intermediate outcomes could also be examined as potential exposures with HIV infection as the outcome.

### Assessment of Validity

We assessed the methodological quality of all included studies using the United Kingdom National Institute for Health and Clinical Excellence (NICE) criteria to assess the quality of reporting in key methodological domains [20]. We included all studies in analyses, irrespective of quality but noted methodological issues that might affect results, such as the lack of an adjusted effect estimate.

### Data Abstraction

We used standardized study selection and data extraction forms. Two teams of two reviewers each (SCF and NB, AMH and MC) assessed the papers for eligibility and extracted data independently. Discrepancies were resolved by discussion or by consultation with a third reviewer (NL). Data items extracted included study population, baseline characteristics, participant retention, and unadjusted and adjusted summary measures with confidence intervals, and lists of variables included in multivariable models. We did not contact authors for additional information. Data were double entered into an Epidata database (Epidata, Odense, Denmark).

### Statistical Analysis

Data were analyzed using Stata, version 10 (StataCorp, College Station, TX). We examined the point estimates and 95% confidence intervals (95% CI) of exposure-outcome pairs in each study according to the study objectives and displayed these in forest plots. We took the rate ratio as the most appropriate effect measure. We assumed that hazard ratios and risk ratios approximated the rate ratio. In nested case control studies, we assumed that the odds ratio approximated the rate ratio if the selection of controls was matched on time and if the analysis took this into account [21]. If the analysis did not take into account matching on time we reported the odds ratio and did not combine these with other effect estimates. We report the original effect measure for single studies and pooled analyses of studies using the same measure. We report pooled analyses from different study designs as summary unadjusted or adjusted effects. Associations reported in more than one publication from the same study were only included once. We examined heterogeneity using the I^2^ statistic, which describes the percentage of total variation across studies that is due to heterogeneity other than chance [22]. An I^2^ value of 50–75% was interpreted as indicating moderate heterogeneity and a value of >75% as showing pronounced between study heterogeneity. If there were more than two reports of the same exposure-outcome pairing from different studies we combined results using a random effects model. If there was strong evidence of statistical heterogeneity we attempted to examine potential explanations in stratified analyses. Funnel plots were examined using statistical methods for detecting asymmetry indicative of small study biases [23].

## Results

**Figure 2:**
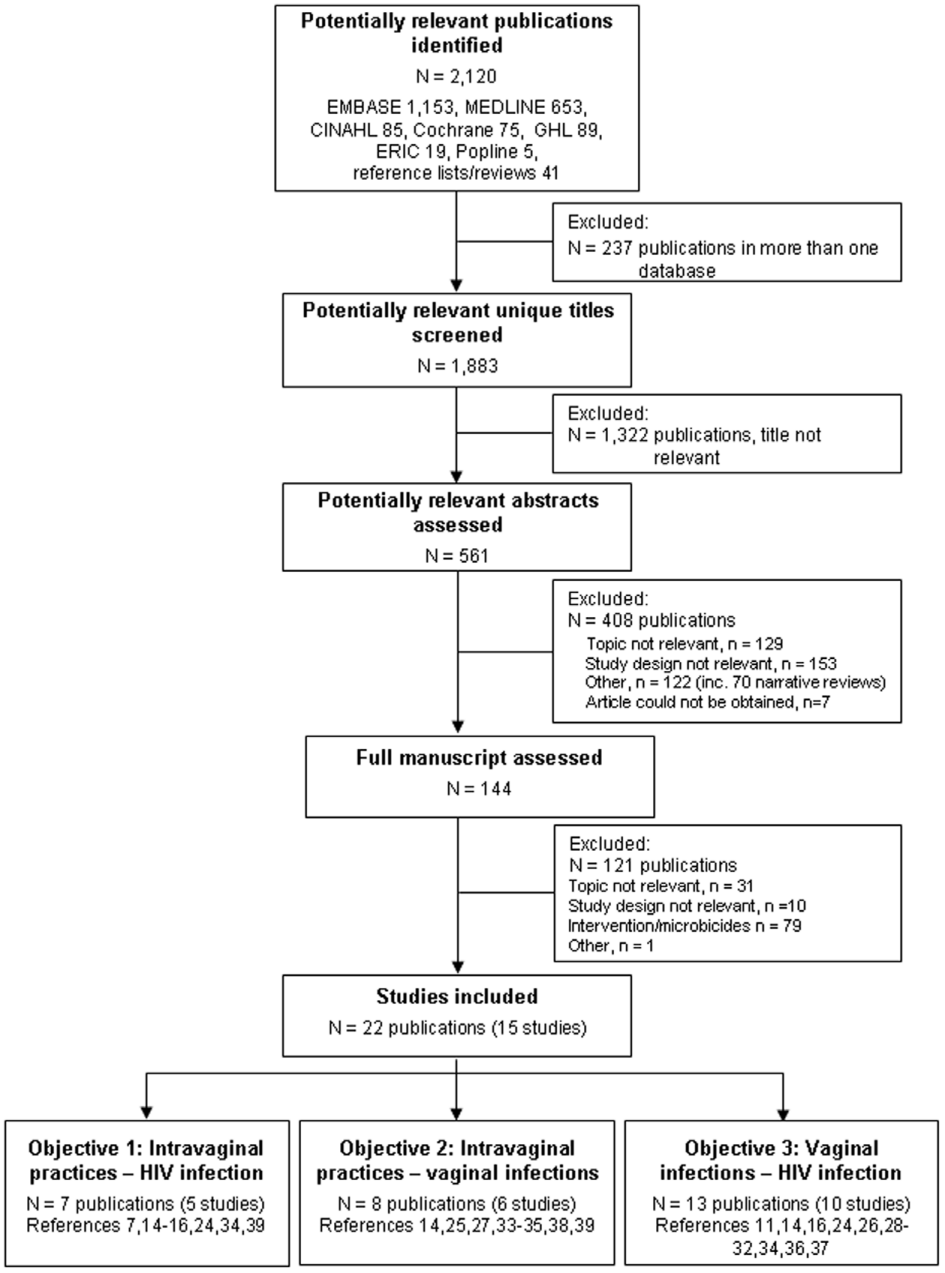
Flow chart of identification and selection of studies for inclusion. Steps followed to identify relevant studies and select those eligible for inclusion in the review. Publications and studies in the box ‘Studies included’ could address multiple objectives and are included in each relevant ‘Objective’ below. EMBASE, Excerpta Medica Database; MEDLINE (Ovid), Medical Literature Analysis and Retrieval System Online; CINAHL, Cumulative Index to Nursing and Allied Health Literature; Cochrane, Cochrane Library (John Wiley); ERIC, Educational Resources Information Centre; GHL, Global Health Library; Popline, Population Information Online.

Our broad search strategies identified 2120 articles (Figure 2); most (70% of unique titles) could be excluded from the title alone. Of 144 full text articles, two thirds of those excluded (79/121) were investigations of microbicides or other ineligible interventions. We included 22 publications from 15 prospective studies (Table 1) [7], [8], [11], [14]–[16], [24]–[39]. One report published after the end of the search period [14] was included because preliminary results had been published in a letter identified by our search strategy [39] and another was in press at the end of the search period [33]. Twenty publications were based on 13 studies in nine sub-Saharan countries (South Africa, N = 4,121 women with follow-up [16], [29], [34], Zimbabwe, N = 3,139 women [8], [14], [30], [38], [39], Kenya, N = 1,335 women [11], [15], [31]–[33], Uganda, N = 2,235 women [14], [39], Tanzania, N = 1,442 women [28], [36], Zambia, N = 724 women [7], [26], Cote d'Ivoire, N = 284 women [24], Malawi, N = 2,538 women [30], [37], Burkina Faso, N = 273 women [35] and the other two publications were from studies conducted in the United States (N = 1,348 women) [25], [27].

**Table 1:** Characteristics of included studies.

First author, publication year [reference no.]	Country	Study design	Population/setting	Number^a^		Duration of follow up (median or woman years)	Vaginal practices described by authors	Objective number^b^
				Enrolled	Follow up			
Ghys, 2001 [24]	Côte d'Ivoire	RCT	Sex worker STD clinic	542	284	318 woman years	Vaginal use of herbs	1, 3
Hawes, 1996 [25]	USA	Cohort	STD clinic attenders	209	182	16 months (median)	Douching	2
Hester, 2003 [26]	Zambia	Case-control	Living with HIV positive partner	Not reported	90	3 months (planned)	Not measured	3
Hira, 1990 [7]	Zambia	Cohort	University hospital, post-partum	1720	634	12 months (planned)	‘Dry sex’ (cloth used to remove vaginal secretions during sex)	1
Hutchinson, 2007 [27]	USA	Cohort	Women at risk of PID	1193	1166	36 months (median)	Douching	2
Kapiga, 2007 [28]	Tanzania	Cohort	Women working in bars	845	689	699 woman years	Not measured	3
Kleinschmidt, 2007 [29]	S Africa	Cohort	Family planning clinics	551	551	491 woman years	Not measured	3
Kumwenda,^c^ 2006 [30]	Malawi, Zimbabwe	Cohort	Family planning or postnatal clinics	2016	2016	2429 woman years	Not measured	3
Martin,^c^ 1998 [31]	Kenya	Cohort	Sex worker STD clinic	953	779	880 woman years	Vaginal cleansing: water, soap/detergent/disinfectant; vaginal drying	3
Martin,^d^ 1999 [32]	Kenya	Cohort	Sex worker STD clinic	953	657	621 woman years		3
McClelland,^d^ 2006 [15]	Kenya	Cohort	Sex worker STD clinic	1496	1270	2877 woman years	Vaginal cleansing: water, soap/detergent/disinfectant; insertion of herbs; use of finger, cloth, douche bag	1
McClelland,^d^ 2007 [11]	Kenya	Cohort	Sex worker STD clinic	1579	1335	3422 woman years	Vaginal cleansing: water, soap/antiseptic; use of finger, cloth; lubricant	3
McClelland,^d^ 2008 [33]	Kenya	RCT placebo	Sex worker STD clinic	154	151	153 woman years		2
Myer,^e^ 2005 [34]	S Africa	Case-control	Cervical cancer screening trial	5110	410	36 months	Wiping inside with water, cloth and/or fingers; sometimes soap or other cleaning agents	1, 2, 3
Myer,^e^ 2006 [16]	S Africa	RCT	Cervical cancer screening trial	4139	3570	4641 woman years	Cloth or fingers, alone or with water; soaps; disinfectants; vinegar; salt water; industrial detergents	1, 3
Nagot, 2007 [35]	Burkina Faso	Cohort	Sex workers	279	273	8.5 months (mean)	‘Vaginal douching’ using soap and water, or other unspecified products	2
Riedner, 2006 [36]	Tanzania	Cohort	Women working in bars	600	753	Up to 27 months	Not measured	3
Taha, 1998 [37]	Malawi	Cohort	Antenatal clinics	1196	1196 antenatal	3.4 months (median antenatal)	Not measured	3
					1169 postnatal	2.5 years (median postnatal)		
van de Wijgert,^f^ 2000a [38]	Zimbabwe	Cohort	Family planning, primary care, postnatal clinics	169	169	6 months (median)	Any vaginal practice (finger cleansing with products other than water, wiping inside vagina >12 times past month, inserting traditional substances >4 times past month	2
van de Wijgert,^f^ 2000b [8]	Zimbabwe	Cohort	Family planning, primary care, postnatal clinics	169	169	6 months (median)		2
van de Wijgert,^g^ 2006 [39]	Uganda, Zimbabwe	Cohort	Family planning, primary care clinics	4531	4531	22 months (mean)	Anything to dry or tighten vagina for sex, anything to clean inside vagina. If yes, prompt about products	1
van de Wijgert,^g^ 2008 [14]	Uganda, Zimbabwe	Cohort	Family planning, primary care clinics	4531	4531	22 months (mean)		1, 2, 3

**Legend:**

PID, pelvic inflammatory disease; RCT, randomized controlled trial; STD, sexually transmitted diseases.

a Number of HIV negative women enrolled; number of women in analyses with at least one follow up visit, or number in case-control study.

b Objective 1, associations between intravaginal practices and incident HIV; objective 2, associations between intravaginal practices and vaginal infections; objective 3, associations between vaginal infections and incident HIV infection.

c Includes 1342 women from Malawi, 674 from Zimbabwe.

d Publications from the same study of an open cohort of sex workers in Mombasa Kenya.

e Publications from the same RCT of a cervical screening intervention in Kayelitsha, South Africa.

f Publications from the same study of the effects of intravaginal practices on vaginal and cervical mucosa.

g Publications from the same study of Hormonal Contraception and Risk of HIV Acquisition in Uganda (2235 women) and Zimbabwe (2296 women).

Of the 22 included publications (Table 1), seven investigated the association between intravaginal practices and HIV acquisition in five studies (objective 1) [7], [14]–[16], [24], [34], [39]; eight explored associations between intravaginal practices and prevalent vaginal infections (objective 2) [14], [25], [27], [33]–[35], [38], [39] in six separate studies; 11 publications in nine studies investigated associations between bacterial vaginosis and HIV acquisition (objective 3) [14], [16], [28]–[32], [34], [36], [37], [39]; nine publications in seven studies reported on the association between trichomoniasis and HIV-1 (objective 3) [11], [16], [24], [26], [28], [29], [31], [34], [37]; and six publications in five different studies reported on the association between vaginal yeast and HIV (objective 3) [14], [26], [28], [31], [37], [39]. The only statistical evidence of small study biases in summary effects was for unadjusted (Egger test p = 0.003) estimates of the association between bacterial vaginosis and HIV. The risk of bias, assessed from the publications, were variable (Table S1). Only one study did not report any multivariable analyses [7], even though relevant adjusted analyses for the associations of interest in this review were often not reported. The reliability of instruments used to record intravaginal practices was less well documented than laboratory exposures and outcomes. The numbers of eligible women who accepted participation were generally poorly reported. Blinding to reduce bias was rarely reported explicitly.

### Types of Intravaginal Practices

Types of intravaginal practices were described in 14 publications from eight studies (Table 1). The most frequently investigated practices involved intravaginal cleansing with liquids. Two studies on vaginal douching from the USA [25], [27] described the use of specific douche applicators and commercial products [27], mainly for cleanliness or hygiene [25]. In sub-Saharan Africa, a wider variety of practices was investigated. Intravaginal cleansing with water or soap and water were investigated most frequently [8], [14]–[16], [31]–[34], [38], [39]. We categorized the practice described as “douching” with soap and water in Burkina Faso as intravaginal washing [35] because no specific douche applicator was mentioned and the use of specific douching applicators was reported to be very uncommon elsewhere in Africa [15]. Other household cleaners and antiseptics [16], [33], vinegar or lemon juice [16] were less commonly included in questionnaires and reported. Where the method used to apply liquids was asked about, fingers were used most commonly [8], [11], [15], [16], [34], [38], [40], with cloth used less often [11], [14]–[16], [33], [34]. Practices aimed at drying or tightening the vagina in preparation for, or during sexual intercourse included: insertion of dry herbs [24], use of a cloth before or during sex [7], or unspecified products [14], [16], [32]. We did not identify any study that specifically investigated the application or ingestion of substances aimed at affecting the vagina or anatomical modifications to the vulva and vagina despite the fact that these practices are also employed to dry and tighten the vagina, or self treat [2]. Combinations of practices, products and methods of application, or ‘any vaginal practice’ were described in some publications [8], [14]–[16], [33], [34], [38].

### Intravaginal Practices and HIV Acquisition

**Figure 3:**
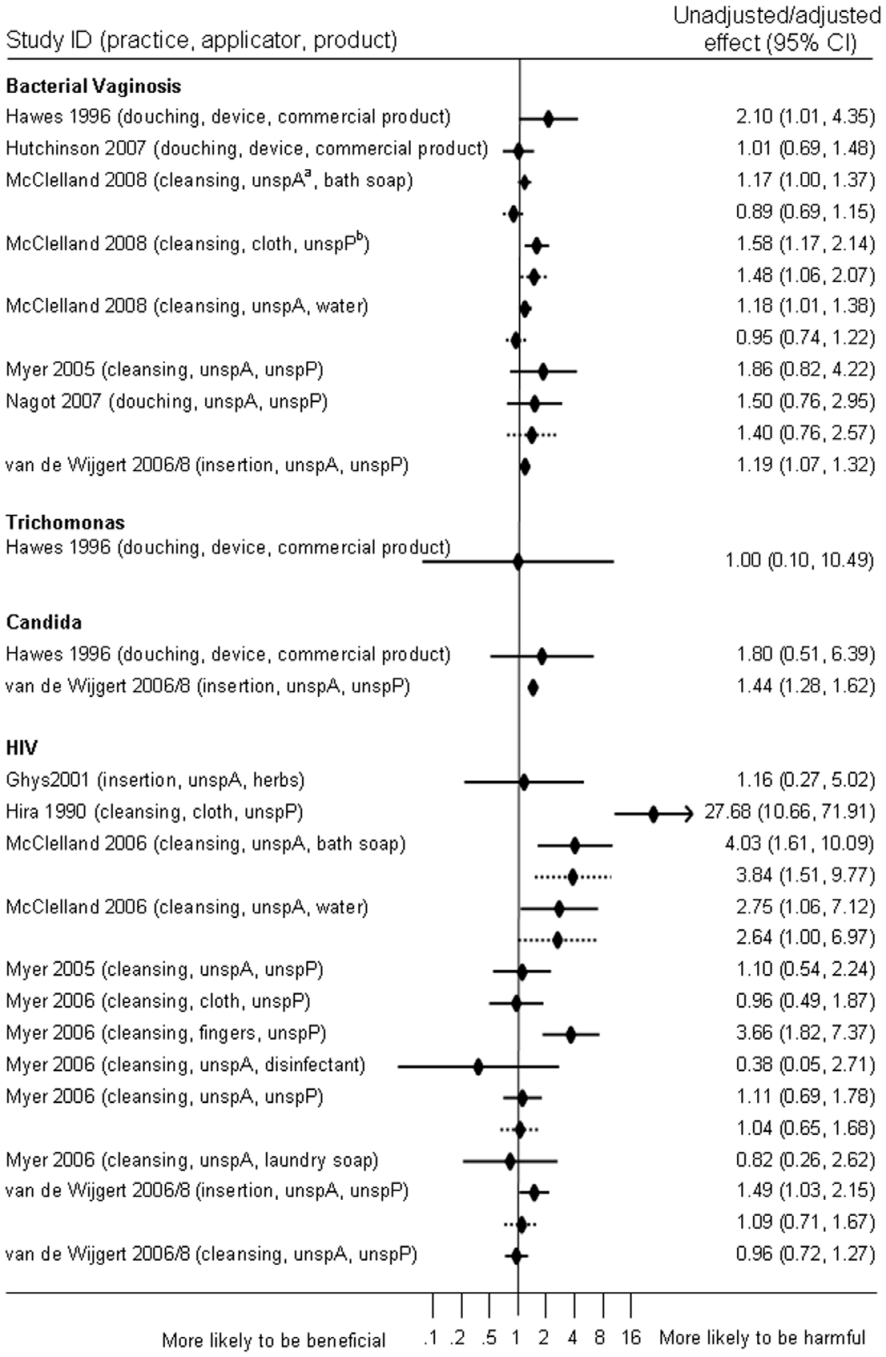
Forest plot of all reported and quantified associations between intravaginal practices and any infection. Forest plot showing unadjusted and/or adjusted effect estimates reported in included studies, according to the infection studied as the outcome. Individual studies can be included more than once if multiple outcomes are reported. If both unadjusted and adjusted effects were reported for the same combination of practice, product and applicator, these are presented with the unadjusted effect estimate above the adjusted effect estimate. No pooled estimates are shown in this plot. Minor differences between effect estimates in the table and those in published papers are possible.

Five studies reported on associations between any kind of intravaginal practice and incident HIV-1 infection in analyses that included 4,169 women with more than 253 HIV infections (number not reported in one study) in seven publications (Table 2, Figure 3 and Table S2) [7], [14]–[16], [24], [34], [39]. All studies were carried out in sub-Saharan Africa, including two in female sex worker populations [15], [24]. There was some evidence of an increased risk of HIV acquisition in unadjusted analyses of these five studies (summary effect 2.65, 95% CI 0.95–7.36, I^2^ 92.0%). Only two studies provided adjusted effect estimates [14], [16]. Three studies reported hazard ratios for the use of intravaginal cleansing with any product [14]–[16]. These suggested harm in unadjusted analyses of all three studies (1.39, 95% CI 0.76–2.54, I^2^ 76.6%) and adjusted analyses including only two studies (adjusted effect estimate 1.88, 95% CI 0.53, 6.69, I^2^ 83.2%) [15], [16] but confidence intervals were wide and heterogeneity high. In two studies reporting on insertion of herbs or unspecified substances to dry and tighten the vagina before sexual intercourse the summary unadjusted effect was 1.47 (95% CI 1.03–2.10, I^2^ 0%) [14], [24]. An adjusted measure was only reported by van de Wijgert et al. [14]. The most marked effect of any practice was reported by Hira, with an increased risk in post-natal women who reported using cloth to wipe out the vagina during intercourse (risk ratio 27.68, 95% CI 10.66–71.92), but loss to follow up from the original cohort was high and there was no multivariable analysis [7]. Myer et al. found no adverse effect in women who reported using cloth inside the vagina (unadjusted hazard ratio 0.96, 95% CI 0.49–1.87) but an increased risk of HIV in women who used fingers to clean the vagina (unadjusted hazard ratio 3.66, 95% CI 1.82–7.37) [16].

**Table 2:** Summary effect estimates for studies reporting associations between intravaginal practices and HIV-1 (objective 1), and intravaginal practices and vaginal infections (objective 2).

Intravaginal practice	Outcome	Unadjusted effect estimate			Adjusted effect estimate		
		(Study ref.)	Summary (95% CI)	I^2^	(Study ref.)	Summary (95% CI)	I^2^
Any vaginal practice^a^	HIV	[7], [14]–[16], [24]	2.65 (0.95, 7.36)	92.0%	[14], [16]	0.87 (0.67, 1.13)	0.0%
Intravaginal cleansing^b^	HIV	[14]–[16]	1.39 (0.76, 2.54)	76.6%	[15], [16]	1.88 (0.53, 6.69)	83.2%
Insertion of substances^c^	HIV	[14], [24]	1.47 (1.03, 2.10)	0.0%	[14]	1.09 (0.71, 1.67)	..
Any vaginal practice^d^	BV	[14], [34], [35]	1.20 (1.09, 1.34)	0.0%	[25], [27], [35]	1.31 (0.87, 1.97)	38.8%
Intravaginal cleansing or douching^e^	BV	[33]–[35]	1.20 (1.03, 1.40)	0.0%	[25], [27], [33]–[35]	1.12 (0.82, 1.54)	49.2%

**Legend:**

a Includes: ‘anything to dry or tighten your vagina for sex’ or ‘anything to clean the inside of your vagina’ [14]; intravaginal washing with water, soap or other substances including detergents and antiseptics [15]; ‘any intravaginal practice reported’ [16]; insertion of herbs [24].

b Includes: ‘anything to clean the inside of your vagina’ [14]; intravaginal washing with soap [15]; ‘any intravaginal practice reported’ [16].

c Includes: ‘anything to dry or tighten your vagina for sex’ [14]; insertion of herbs [24].

d Includes: ‘anything to dry or tighten your vagina for sex’ or ‘anything to clean the inside of your vagina’ [14]; ‘douching for cleanliness’ [25]; ‘douching’ [27]; ‘wiping inside the vagina with water, cloth and/or fingers and sometimes with soap or other cleaning agents as part of regular hygiene [34]; ‘vaginal douching using only soap and/or water’ [35].

### Intravaginal Practices and Vaginal Infections

Associations between intravaginal practices and incident bacterial vaginosis or disturbance of intravaginal flora were reported in six studies involving 6,503 women in analyses [14], [25], [27], [33]–[35] (Table 2, Figure 3 and Table S3). The summary measure of effect in unadjusted analyses of three studies suggested an association between intravaginal douching or washing and bacterial vaginosis in women with normal flora at baseline (1.20, 95% CI 1.03–1.40, I^2^ 0%) [33]–[35]. This was attenuated in an adjusted summary effect that included different studies (1.12, 95% CI 0.82–1.54, I^2^ 49.2%) [25], [27], [33], [35]. In Zimbabwe and Uganda, insertion of substances to dry or tighten the vagina before sexual intercourse was associated with incident bacterial vaginosis in univariable but not multivariable analyses [14]. Trichomoniasis and candidiasis were studied as outcomes following intravaginal practices in only two studies [14], [25]. Hawes et al. found no statistical evidence for an association between douching and trichomonas or candidiasis [25], whilst insertion of substances for drying or tightening the vagina [14] was associated with vaginal candida in univariable analyses (Figure 3).

### Vaginal Infections and Incident HIV Infection

**Figure 4:**
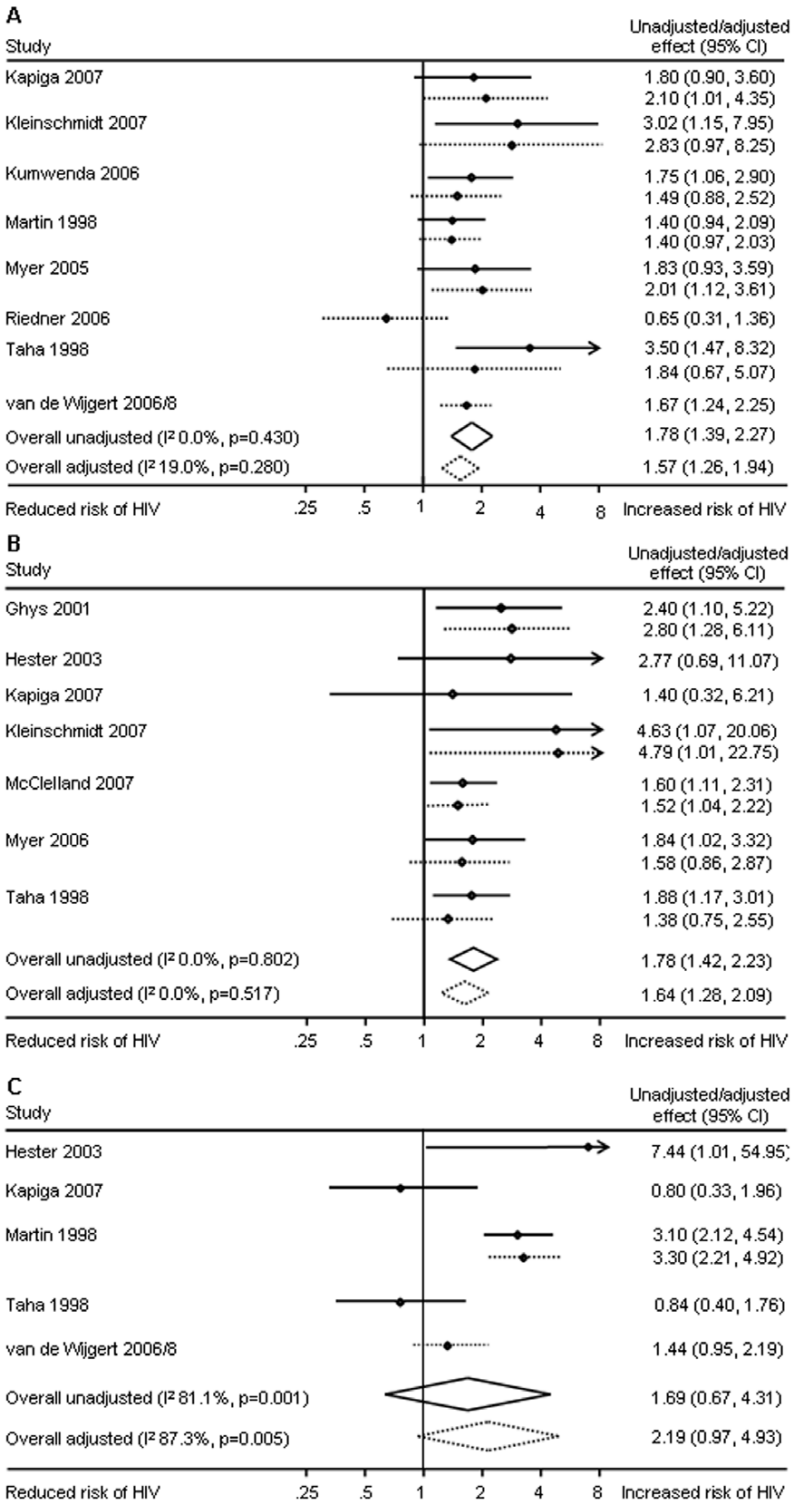
Meta-analyses of studies reporting associations between vaginal infections and HIV, unadjusted and adjusted effect estimates. Panel A: Bacterial vaginosis is associated with incident HIV infection. Eight studies contribute to the pooled adjusted effect estimate with little between study heterogeneity. Panel B: *Trichomonas vaginalis* infection is associated with incident HIV infection. Five studies contribute to the pooled adjusted effect estimate with no between study heterogeneity. Panel C: Candida or other yeast infections are not consistently associated with incident HIV infection. Two studies, with differing results contribute to the pooled adjusted effect estimate. Minor differences between effect estimates in the table and those in published papers are possible.

There was strong and consistent evidence of associations between bacterial vaginosis and HIV and between trichomoniasis and HIV in both unadjusted and adjusted analyses (Figure 4). Evidence of an association between vaginal yeast infections and HIV infection was based on fewer studies and showed heterogeneous results in unadjusted (summary effect 1.69, 95% CI 0.67–4.31, I^2^ 81%) and adjusted analyses (summary effect 2.19, 0.97–4.94, I^2^ 87%).

## Discussion

This systematic review and meta-analysis of 22 publications from 15 studies in sub-Saharan Africa and the USA found no evidence that intravaginal practices reduce the incidence of vaginal infections or HIV. There was inconclusive statistical evidence about the effect of intravaginal practices on women's risk of acquiring HIV infection; the direction of summary measures of associations suggested harm, but confidence intervals were wide and there were high levels of heterogeneity. There was evidence of an association between intravaginal washing and douching practices and bacterial vaginosis in unadjusted but not adjusted analyses. There was strong statistical evidence that bacterial vaginosis and trichomoniasis increase women's risk of acquiring HIV infection.

The main strength of this study was the comprehensive search for studies examining all stages of a potential pathway linking intravaginal practices and HIV infection. Our systematic searches of multiple databases without language restrictions are unlikely to have missed important prospective studies of intravaginal practices and HIV infection. The specific keywords used to identify individual vaginal infections might, however, have failed to identify studies that were indexed only under the general term ‘sexually transmitted diseases’. We also attempted to distinguish the effects of different intravaginal practices using categories based on empirical research that describe practices according to the type of practice, method of application and product [2]. Across all included studies, however, measurement of vaginal practices, grouping of practice types and the period of recall varied, limiting direct comparisons between studies and identification of specific potential harms. We were only able to estimate the effects for broad categories of practice such as intravaginal cleansing with any product, or use of any product for drying and tightening the vagina for sex. Between study heterogeneity in study populations and the practices included therefore limit interpretation if combinations of practices or products with different effects dilute the associations. There was some evidence for small trials bias in one analysis only; the unadjusted association between bacterial vaginosis and HIV. However, this association persisted in the adjusted analysis for which there was no statistical evidence of such biases.

One previous review examined associations between intravaginal practices, vaginal infections and HIV [13]. Myer and colleagues identified and included both cross-sectional and prospective studies published up to 2004 and found strong evidence of an association between any intravaginal practices and prevalent HIV infection and when combining unadjusted and adjusted estimates. The association between intravaginal practices and HIV infection in three studies suggested a harmful effect but confidence intervals were wide (summary estimate 3.85, 95% CI 0.52, 28.27). In this review that includes several more recently published studies we only included prospective studies enrolling HIV seronegative women so that the temporal sequence of events between intravaginal practices and HIV infection was clear. We also studied a wider range of potential intermediate infections, including vaginal yeast and trichomoniasis. Few other studies have examined all links in this putative pathway. In a large study conducted in Uganda and Zimbabwe, van de Wijgert et al. concluded that the use of all intravaginal practices combined did not increase the risk of vaginal infections or HIV [14]. In our review, we also found some evidence in unadjusted analyses that any vaginal practice, products inserted to dry or tighten the vagina for sex, and products used for intravaginal cleansing increased women's risk of acquiring HIV infection. Adjusted summary measures were difficult to interpret because they included fewer studies, some of which were different from those in the unadjusted analyses.

Our review showed no evidence that the intravaginal practices investigated protect against the development of bacterial vaginosis, or acquisition of other vaginal infections or HIV, even though previous studies have found that women report that improving genital hygiene and treating symptoms are common motivations for these practices [2], [17], [41]. Women might be using intravaginal practices to assert control over their sexuality and health without realizing that the products and methods of application might not be beneficial. As intravaginal washing practices are common in sub-Saharan Africa, even small increases in risk for HIV acquisition could mean a substantial proportion of HIV infections are attributable to these practices. Grimley et al. showed that a counseling intervention reduced douching in young women in the USA [42]. Further trials that attempt to modify intravaginal practices that are evaluated with biological outcomes would show whether the incidence of vaginal infections and HIV can also be reduced.

This study showed strong and consistent evidence that trichomoniasis increases women's risk of acquiring HIV [43]. The strength of the association is similar to that for the association between bacterial vaginosis and incident HIV, which is now well documented [10] and confirmed in our analyses. Trichomonas can cause intense vaginal mucosal disruption but subclinical inflammation in asymptomatic infections might also be sufficient to increase HIV transmission. Trichomonas is sexually transmitted but re-infection is common despite partner notification [44]. Another potential route of re-infection might be from cloths, reported as being used to wipe the vagina for washing [11] or during sex [7], if trichomonads remain viable on damp cloths. The association between vaginal practices and trichomonas merits further study since we found only one small study from the USA, which found no association [25]. Modification to such practices might be possible and McClelland et al. found modest evidence that periodic presumptive treatment reduced the incidence of trichomonas [40].

The findings of this study have implications for HIV prevention research, particularly in the field of vaginal microbicide development. A sub-group analysis of one trial found some evidence of benefit of 0.5% PRO 2000/5 gel [45]. Several other phase 3 trials, however, found that microbicides with favorable *in vitro* activity and safety profiles did not prevent, and might have increased, the risk of HIV infection [46], [47]. Intravaginal practices could interfere with the intended effects of chemical microbicides by washing them out, diluting them, or interacting chemically to reduce the expected efficacy. Cervicography has been used to show how detergents damage and disrupt intravaginal mucosa [48]. Alternatively, previously unidentified mechanisms, such as disruption of tight junctions in the epithelial barrier, could increase the risk of HIV transmission [49]. Toxicological studies could help to find out whether or not soaps and other products that are used intravaginally inactivate microbicides. Qualitative research would help us to understand whether or not the timing of or motivations for intravaginal practices might be interfering with microbicide delivery. Further individual studies of the association between intravaginal practices and HIV infection will require very large numbers of participants and prolonged repeated follow up visits, however, so that the effects of individual practices, applicators and products can be examined appropriately. An alternative would be to pool data from existing studies, create common variables across studies and conduct individual person data meta-analysis. In summary, the current evidence shows that intravaginal practices do not appear to protect women from vaginal infections or HIV and some might be harmful.

## Supporting Information

Table S1 Assessment of reporting of features related to the risk of bias. *** criterion well-covered; ** criterion adequately addressed; * criterion poorly addressed; - criterion not addressed or not reported; VI vaginal infection; VP intravaginal practice; HIV - human immunodeficiency virus infection; where more than one of our study objectives was addressed (see Table 1), exposure and outcome that were assessed are in brackets. Items selected from reference 20.
(0.07 MB DOC)

Table S2 Studies reporting associations between intravaginal practices and incident HIV infection (objective 1). BV, bacterial vaginosis; CT, *Chlamydia trachomatis*; HSV2, herpes simplex virus type 2; NG, *Neisseria gonorrhoeae*; NR, not reported; wy, woman years of follow up; WBC, white blood cells.
(0.07 MB DOC)

Table S3 Studies reporting associations between intravaginal practices and vaginal infections (objective 2). BV, bacterial vaginosis; HSV2, herpes simplex virus type 2; H2O2, hydrogen peroxide producing *Lactobacillus spp.*; NR, not reported; TV, *Trichomonas vaginalis*; WBC, white blood cells.
(0.12 MB DOC)
