# Supplementary material for: Intravaginal Practices, Vaginal Infections and HIV Acquisition: Systematic Review and Meta-Analysis
Source: PLoS One. 2010 Feb 9;5(2):e9119. doi: 10.1371/journal.pone.0009119 (PMC2817741; doi:10.1371/journal.pone.0009119)
Supplement: Table S1 — Assessment of reporting of features related to the risk of bias. *** criterion well-covered; ** criterion adequately addressed; * criterion poorly addressed; - criterion not addressed or not reported; VI vaginal infection; VP intravaginal practice; HIV - human immunodeficiency virus infection; where more than one of our study objectives was addressed (see Table 1), exposure and outcome that were assessed are in brackets. Items selected from reference 20. (0.07 MB DOC) [file pone.0009119.s001.doc]

|  | **Comparable populations (exposed/un-exposed, or case/control)** | **Participation rate amongst all eligible reported** | **Include only incident outcomes in analysis** | **Analyses dealing with main confounders** | **Reliable exposure measure^a^** | **Outcome measure is valid and reliable^a^** | **Blinded outcome assessment explicit** | **Loss to follow up, %** | **Compare lost with not lost to follow up** |
| --- | --- | --- | --- | --- | --- | --- | --- | --- | --- |
| Ghys, 2001 [24] | *** | * | ** | ** | (VP) – | (HIV) ** | – | 53 | yes |
| Hawes, 1996 [25] | – | – | *** | ** | (VP) ** | (VI) *** | – | 13 | no |
| Hester, 2003 [26] | * | * | ** | * | (VI) * | (HIV) ** | – | 11 | no |
| Hira, 1990 [7] | – | – | *** | – | (VP) – | (HIV) ** | – | 63 | unclear |
| Hutchinson, 2007 [27] | *** | *** | ** | ** | (VP) – | (VI) *** | – | 2 | unclear |
| Kapiga, 2007 [28] | ** | ** | *** | *** | (VI) ** | (HIV) *** | – | 19 | yes |
| Kleinschmidt, 2007 [29] | * | * | *** | ** | (VI) *** | (HIV) ** | – | 12 | no |
| Kumwenda, 2006 [30] | *** | * | * | * | (VI) ** | (HIV) *** | – | 17 | unclear |
| Martin, 1998 [31] | *** | ** | *** | ** | (VI) *** | (HIV) *** | * | 18 | yes |
| Martin, 1999 [32] | ** | – | – | ** | (VI) *** | (HIV) *** | – | – | no |
| McClelland, 2006 [15] | ** | * | ** | ** | (VP) – | (HIV) ** | – | 15 | yes |
| McClelland, 2007 [11] | *** | * | *** | ** | (VI) ** | (HIV) ** | – | 16 | yes |
| McClelland, 2008 [33] | *** | ** | * | ** | (VP) ** | (VI) *** | – | 2 | no |
| Myer, 2005 [34] | *** | ** | *** | *** | (VI) ** | (HIV) ** | *** | 21 | Yes |
| Myer, 2006 [16] | *** | * | ** | *** | (VP) ** | (HIV) ** | – | 14 | yes |
| Nagot, 2007 [35] | *** | * | * | ** | (VP) – | (VI) *** | – | - | no |
| Riedner, 2006 [36] | *** | * | *** | ** | (VI) *** | (HIV) ** | – | 23 | yes |
| Taha, 1998 [37] | * | – | – | *** | (VI) ** | (HIV) ** | – | – | unclear |
| van de Wijgert, 2000a [38] | * | * | ** | ** | (VI) ** | (VP) *** | – | 8 | no |
| van de Wijgert, 2000b [8] | * | – | – | ** | (VI) ** | (VP) *** | – | 20 | yes |
| van de Wijgert, 2006/8 [39, 14] | * | – | * | *** | (VP) ** | (HIV) *** | – | 10 | yes |
