# Supplementary material for: Intravaginal Practices, Vaginal Infections and HIV Acquisition: Systematic Review and Meta-Analysis
Source: PLoS One. 2010 Feb 9;5(2):e9119. doi: 10.1371/journal.pone.0009119 (PMC2817741; doi:10.1371/journal.pone.0009119)
Supplement: Table S2 — Studies reporting associations between intravaginal practices and incident HIV infection (objective 1). BV, bacterial vaginosis; CT, Chlamydia trachomatis; HSV2, herpes simplex virus type 2; NG, Neisseria gonorrhoeae; NR, not reported; wy, woman years of follow up; WBC, white blood cells. (0.07 MB DOC) [file pone.0009119.s002.doc]

| **Study** | **Intravaginal Practice** | **Applicator or product** | **Exposed, n (woman years)** | **Events Exposed** | **Unexposed, n (woman years)** | **Events Unexposed** | **Reported Summary Measure** | **Unadjusted ratio (CI)** | **Adjusted ratio (CI)** | **Variables adjusted for** |
| --- | --- | --- | --- | --- | --- | --- | --- | --- | --- | --- |
| Ghys, 2001 [24] | Insertion | Herbs | 24 (26) | 2 | 233 (241) | 16 | Rate ratio | 1.16 (0.27-5.06) | Not reported |  |
| Hira, 1990 [7] | Intravaginal cleansing | Cloth | 36 | 10 | 598 | 6 | Risk ratio | 27.68 (10.66-71.92) | Not reported |  |
| McClelland, 2006 [15] | Intravaginal cleansing | Any product | 2605 | 217 | 272 | 5 | Rate ratio | 4.52 (1.66-12.27) | Not reported |  |
|  | Intravaginal cleansing | Water alone | NR | 45 | NR | 5 | Hazard ratio | 2.75 (1.06-7.10) | 2.64 (1.00-6.97) | Demographic factors, sexual behavior, sexually transmitted infections |
|  | Intravaginal cleansing | Soap and water | NR | 172 | NR | 5 | Hazard ratio | 4.03 (1.61-10.10) | 3.84 (1.51-9.77) |
| Myer, 2006 [16] | Any vaginal practice | Any product/ applicator | 945 | 24 | 2625 | 61 | Hazard ratio | 1.11 (0.69-1.78) | 1.04 (0.65-1.68) | Age, marital status, TV GC and/or CT, new sex partners past 5m |
|  | Intravaginal cleansing | Fingers +/- water | 101 | 9 | 2625 | 61 | Hazard ratio | 3.66 (1.82-7.37) | Not reported |  |
|  | Intravaginal cleansing | Cloth +/- water | 474 | 10 | 2625 | 61 | Hazard ratio | 0.96 (0.49-1.87) | Not reported |  |
|  | Intravaginal cleansing | Household/laundry soap | 158 | 3 | 2625 | 61 | Hazard ratio | 0.82 (0.26-2.62) | Not reported |  |
|  | Intravaginal cleansing | Household disinfectant | 121 | 1 | 2625 | 61 | Hazard ratio | 0.38 (0.05-2.43) | Not reported |  |
| van de Wijgert, 2006 [39], 2008 [14] | Any intravaginal practice | Any applicator | 3045 | NR | 1486 | NR | Hazard ratio | 0.96 (0.72-1.27) | 0.81 (0.59-1.10) | BV, yeast, WBC, country, contraception, births, behavior, STI, HSV2 |
|  | Intravaginal cleansing | Unspecified applicator/ product | NR | NR | NR | NR | Hazard ratio | 0.96 (0.72-1.27) | No association in multivariable model (values not reported) | |
|  | Insertion of solids | Unspecified applicator | NR | NR | NR | NR | Hazard ratio | 1.49 (1.03-2.15) | 1.09 (0.71-1.67) | BV, yeast, WBC, country, contraception, births, behavior, STI, HSV2 |
