# Supplementary material for: Intravaginal Practices, Vaginal Infections and HIV Acquisition: Systematic Review and Meta-Analysis
Source: PLoS One. 2010 Feb 9;5(2):e9119. doi: 10.1371/journal.pone.0009119 (PMC2817741; doi:10.1371/journal.pone.0009119)
Supplement: Supplementary file 3 [file pone.0009119.s003.doc]

Study	Vaginal Practice	Applicator or product	Exposed (events/woman years)	Unexposed (events/woman years)	Reported measure	Unadjusted (95%CI)	Adjusted (95%CI)	Variables controlled for
**Outcome: Bacterial Vaginosis**								
Hawes, 1996 [25]	Douching	Commercial douche	NR	NR	Hazard ratio	NR	2.10 (1.00-4.30)	H2O2, lactobacilli, prior BV, new partner, age>20, ethnicity
Hutchinson, 2007 [27]	Douching	Commercial douche	NR	NR	Hazard ratio	NR	1.01 (0.69-1.48)	Ethnicity, education, sex partners, Gram stain score, history of STD, BV
McClelland, 2008 [33]	Intravaginal washing	Water	NR	NR	Hazard ratio	1.18 (1.01-1.38)	0.95 (0.74-1.22)	Age, years of sex work, education, bar work, tobacco, alcohol, washing frequency, sex partners, unprotected sex, contraception, TV, candida, H2O2, cervicitis
	Intravaginal washing	Soap or antiseptic	NR	NR	Hazard ratio	1.17 (1.00-1.38)	0.89 (0.69-1.15	
	Intravaginal washing	Cloth	NR	NR	Hazard ratio	1.58 (1.17-2.14)	1.48 (1.06-2.08)	
Myer, 2005 [34]	Any vaginal practice	Cloth and/or fingers with water, soap,or other cleaning agents	NR	NR	Rate ratio	1.86 (0.82-4.23)	No association" (values and variables adjusted for not reported	
Nagot, 2007 [35]	Intravaginal washing	Soap and water	169/757	6/44	Rate ratio	1.5 (0.8-3.1)	1.4 (0.76-2.57)	Sex partners, hormonal contraception, TV, HSV2, HIV
van de Wijgert, 2008 [16]	Any vaginal practice	Unspecified products	NR	NR	Hazard ratio	"No association" (values and variables adjusted for not reported)		
	Drying or tightening	Unspecified products	NR	NR	Hazard ratio	1.19 (1.07-1.32)	No association" (values and variables adjusted for not reported	
**Outcome: Trichomonas vaginalis**								
Hawes, 1996 [25]	Douching	Commercial douche	NR	NR	Hazard ratio	NR	1.00 (0.10-11.00)	H2O2 neg, +/- lactobacilli, prior infection, new partner, age>20, non-white,
**Outcome: Yeast vaginitis**								
Hawes, 1996 [25]	Douching	Commercial douche	NR	NR	Hazard ratio	NR	1.80 (0.50-6.30)	H2O2 neg, +/- lactobacilli, prior infection, new partner, age>20, non-white,
van de Wijgert, 2008 [16]	Any vaginal practice	Unspecified products	NR	NR	Hazard ratio	0.93 (0.84-1.02)	No association" (values and variables adjusted for not reported	
	Drying or tightening	Unspecified products	NR	NR	Hazard ratio	1.44 (1.28-1.63)	No association" (values and variables adjusted for not reported	
